# Laboratory evaluations of the immediate and sustained efficacy of lotilaner (Credelio™) against four common species of ticks affecting dogs in North America

**DOI:** 10.1186/s13071-017-2476-y

**Published:** 2017-11-01

**Authors:** Martin Murphy, Roberto Garcia, Daniela Karadzovska, Daniela Cavalleri, Dan Snyder, Wolfgang Seewald, Theresa Real, Jason Drake, Scott Wiseman, Steve Nanchen

**Affiliations:** 1 Elanco Animal Health, Mattenstrasse 24a, 4058 Basel, Switzerland; 2 Elanco Animal Health, 2500 Innovation Way, Greenfield, IN 46140 USA; 3 Elanco Animal Health, Yarrandoo, NSW Australia; 4 Elanco Animal Health, Southampton, Hants UK

**Keywords:** Lotilaner, Credelio, Ticks, *Ixodes*, *Rhipicephalus*, *Amblyomma*, *Dermacentor*, Dog

## Abstract

**Background:**

Effective control of tick infestations on dogs is important to reduce the risk of transmission of bacterial, viral, and protozoal pathogens. Laboratory studies were initiated to determine the efficacy of lotilaner against common ticks infesting dogs in the United States.

**Methods:**

Eight studies investigated the efficacy of lotilaner against ticks. In two studies dogs were infested with both *Dermacentor variabilis* and *Rhipicephalus sanguineus*: one additional study was completed for each of these species. Two studies assessed infestations with *Amblyomma americanum* and two with *Ixodes scapularis*. In all studies, dogs were ranked and blocked by counts from pre-treatment infestations and randomly allocated, at least eight per group, to be treated orally with lotilaner (minimum dose rate 20 mg/kg), or to be untreated controls. Treatments were administered on Day 0, within 30 min after dogs were fed. In all studies, infestations were performed with 50 adult ticks on Days -2, 7, 14, 21 and 28, and also on Day 35 for *R. sanguineus*, *D. variabilis* and *I. scapularis.* Tick counts were completed 48 h after treatment or after each subsequent challenge. An adequate infestation was defined as at least 25% of the infestation dose recovered from each of at least six control animals at each evaluation. Efficacy calculations for the primary objective were based on geometric means.

**Results:**

In all studies, lotilaner was 100% effective against existing infestations. For post-treatment assessments, on only two occasions did efficacy fall below 99%: in one *D. variabilis* study efficacy was 98.0% on Day 35 and in one *I. scapularis* study efficacy on Day 16 was 98.4%. Only mild and transient adverse events were observed, and none were considered to be related to treatment.

**Conclusion:**

Lotilaner was completely effective against existing infestations with four common species of ticks, *D. variabilis, R. sanguineus, A. americanum* and *I. scapularis,* that affect dogs in North America, with at least 4 weeks efficacy of 98.0% or more against subsequent challenge infestations. These results show that lotilaner is a highly effective isoxazoline that offers sustained efficacy against ticks through and beyond the one-month end-of-dose treatment interval.

**Electronic supplementary material:**

The online version of this article (10.1186/s13071-017-2476-y) contains supplementary material, which is available to authorized users.

## Background

Effective control of tick infestations on dogs is important to reduce the risk of transmission of a variety of bacterial, viral, and protozoal pathogens. Ticks are also a source of irritation at the site of their attachment, and when attached to a host can be difficult to remove. Improper removal can lead to longer term inflammatory consequences [1]. Year-round tick control is, therefore, important, and products that are used should be effective against attached ticks and provide post- treatment protection that will limit the risk of prolonged attachment from new challenges, particularly as acaracidal levels may decline toward the end of the between-dose period (end-of-dose).

Four species of ticks that commonly infest dogs in North America are *Dermacentor variabilis, Rhipicephalus sanguineus*, *Amblyomma americanum* and *Ixodes scapularis.* Until recently, control of these species has been dependent on topically applied chemicals or on the use of collars that release chemicals onto the treated animal’s hair coat. Both measures carry some risk of owner exposure to the pesticide, or of oral ingestion by the treated or in-contact pets [2]. A limitation of externally applied products is the potential for climatic factors and water exposure to reduce ongoing efficacy [3]. Additionally, to ensure effectiveness, topical products must be carefully applied by the owner.

Discovery of the novel family of compounds, the isoxazolines, has allowed the development of orally administered products with the potential to provide knockdown of infesting fleas and ticks and activity against post-treatment challenge [4–6]. Lotilaner is a newly developed isoxazoline that has been shown to have rapid absorption and a long half-life following oral administration of a flavoured chewable tablet to dogs [7]. This rapid absorption of lotilaner has been shown to translate into a rapid onset of activity against fleas with high efficacy sustained through 35 days after a single treatment [8, 9]. To determine if lotilaner would provide equivalent sustained efficacy against ticks, eight studies were undertaken in which treated dogs were challenged with one or more of each of four common species of ticks that infest dogs in North America.

The objective of each study was to determine the efficacy and safety of lotilaner flavoured chewable tablets when dosed orally to dogs at a minimum dose rate of 20 mg/kg against infestations of ticks present at the time treatment was administered, or against new infestations at 28 to 35 days post-treatment. In two studies dogs were infested with both *D. variabilis* and *R. sanguineus* (Studies 1 and 2) and there was one additional study for each of those species (Studies 3 and 4, respectively)*.* Two studies were conducted for each of *A. americanum* (Studies 5 and 6) and *I. scapularis* (Studies 7 and 8). In the *A. americanum* studies, too few ticks were available to allow meaningful challenges beyond Day 28. All studies were single-center, investigator/assessor-blinded, and randomised with eight or ten healthy dogs per study group.

## Methods

Studies were completed at laboratories in the United States (Arkansas, Georgia, and Texas), Ireland and Switzerland. Protocols were prepared in consideration of the recommendations set forth by the World Association for the Advancement of Veterinary Parasitology guidelines for evaluation of the efficacy of parasiticides for the treatment, prevention and control of flea and tick infestations of dogs and cats [10]. Studies were conducted according to the principles of Good Clinical Practices and Good Laboratory Practices (GLP) for Non-Clinical Laboratory Studies [11, 12].

### Animals and housing

Seven of the eight studies enrolled 16 Beagle dogs; 20 dogs were enrolled in Study 6. Dogs were at least 6 months old, and weighing from 6.7 to 20.0 kg. Before enrollment, all dogs had been acclimated to the study facility. To qualify for inclusion each dog was required to be healthy and to demonstrate susceptibility to tick infestation based on retention of at least 25% of a tick challenge conducted, depending on the study, from Day -14 to Day -7. Dogs were excluded if pregnant or lactating, or if they had been exposed to ectoparasiticide treatments for 8 weeks to 6 months before study enrollment, depending on the duration of activity of the product used. All dogs were individually housed during tick challenge periods, had access to water *ad libitum* and were fed a commercially available high quality complete canine diet according to each facility’s standard procedure.

### Tick infestations and counts

Tick infestations were completed by gentle application to a dog’s dorsal or lateral rump or abdomen, with either sedation with medemotidine, or manual restraint without sedation for up to a minute to allow ticks to crawl into the dog’s hair. For each study, infestations were completed by applying approximately 50 ticks of the relevant species (approximately male:female ratio, 1:1) on Day -2, and post-treatment on Days 7, 14, 21, and 28, and also on Day 35 for *R. sanguineus*, *D. variabilis* and *I. scapularis*.

Ticks of each species tested were obtained from laboratory-maintained colonies. All colonies were initiated with field isolates and had been refreshed at yearly or two-yearly intervals with additional field-caught ticks from different areas. In one *I. scapularis* study, ticks were field-collected by flagging vegetation in South Carolina. Captured ticks were maintained in glass vials with moist filter paper and placed in a chamber which provided a relative humidity and temperature of approximately 98.5% and 11.1 °C, respectively.

Tick removals from dogs and counts of live ticks were completed at approximately 48 h post-treatment and approximately 48 h after each subsequent infestation. Study tick count procedure consisted of a thorough examination of all body areas by palpation with the finger tips (thumb counting) first to locate and count the attached/free ticks followed by combing the animal’s coat to remove all ticks. Ticks were considered alive if legs reacted to a tactile or stimulus or to exhaled air (carbon dioxide) and were considered dead if they did not.

### Randomization and treatment

Between one and 2 weeks before administration of study treatment, a pre-treatment infestation was performed and ticks removed and counted 48 h later. Counts of living, attached ticks were used for ranking, blocking and randomising each dog to one of the two treatment groups.

In each study, dogs in one group received lotilaner chewable flavoured tablets, administered orally as close as possible to the minimum recommended dose rate of 20 mg/kg, without underdosing. Based on the available tablet sizes of 56.25 mg, 112.5 mg and 225 mg dogs were dosed with the best tablet combination to reach this target dose rate, according to their body weight. Dogs in the other group were untreated or sham-treated negative controls to facilitate blinding of study staff. All dogs had consumed at least one-third of the daily ration within 30 min before dosing. On Day 0, treatments were administered directly into each dog’s mouth to ensure the targeted dose was delivered.

Clinical observations were made before dosing and at approximately one, six and 8 h (± 15 min) post-dose to observe for adverse events. Post-dose observations of each dog’s health were performed at least once daily through to the end of the study.

### Efficacy assessment

The individual dog was the experimental unit. The efficacy of lotilaner was calculated separately for each tick species by comparing geometric, and arithmetic mean counts in the lotilaner group with those of the untreated control group. Abbott’s formula was used to calculate efficacy.

$$ \mathrm{Percent}\  \mathrm{efficacy}=100\times \left(\mathrm{Mc}-\mathrm{Mt}\right)/\mathrm{Mc} $$

where Mc is the mean number of live ticks (per species on animals) in the untreated control group, and Mt. is the mean number of live ticks on animals in the treated group.

There were separate calculations for each tick species at each counting time point. Since the calculation of the geometric mean involved taking the logarithm of the tick count of each animal, for any tick counts that were equal to zero a one (1) was added to the count for every animal in every group. If a one (1) was added to the tick count, this constant (1) was subtracted from the resultant calculated geometric mean before calculating percent efficacy.

Lotilaner was considered effective at a given time point if the following criteria were met for the treatment group and tick species at that time point: (i) An adequate infestation was achieved in control group dogs. An adequate infestation was defined as at least 25% of the infestation dose (i.e. ≥ 12 ticks) were recovered from each of at least 6 control animals at each evaluation; (ii) There was a statistically significant difference (two-sided level, *P* < 0.05) in geometric mean tick counts between the treated group and the untreated control group, with a significantly lower number of live (attached and unattached) ticks in the treated group compared to the negative control; (iii) The treated group had a calculated efficacy of at least 90%.

### Translation

Spanish translation of the article is available in Additional file 1.

## Results

An adequate infestation (at least six untreated control dogs with an attachment rate greater than 25% for the tested tick species, and an average control group infestation rate greater than 25%, or 12 ticks) was demonstrated in all but three studies. The exceptions were in control-group infestation rates in a *D. variabilis* study (Study 2, on Day 9), a *R. sanguineus* study (Study 3, Day 2), and in the study with field-collected *I. scapularis* (Study 8, Day 16). At each of these assessments, there were no live ticks on lotilaner-treated dogs.

**Fig. 1:**
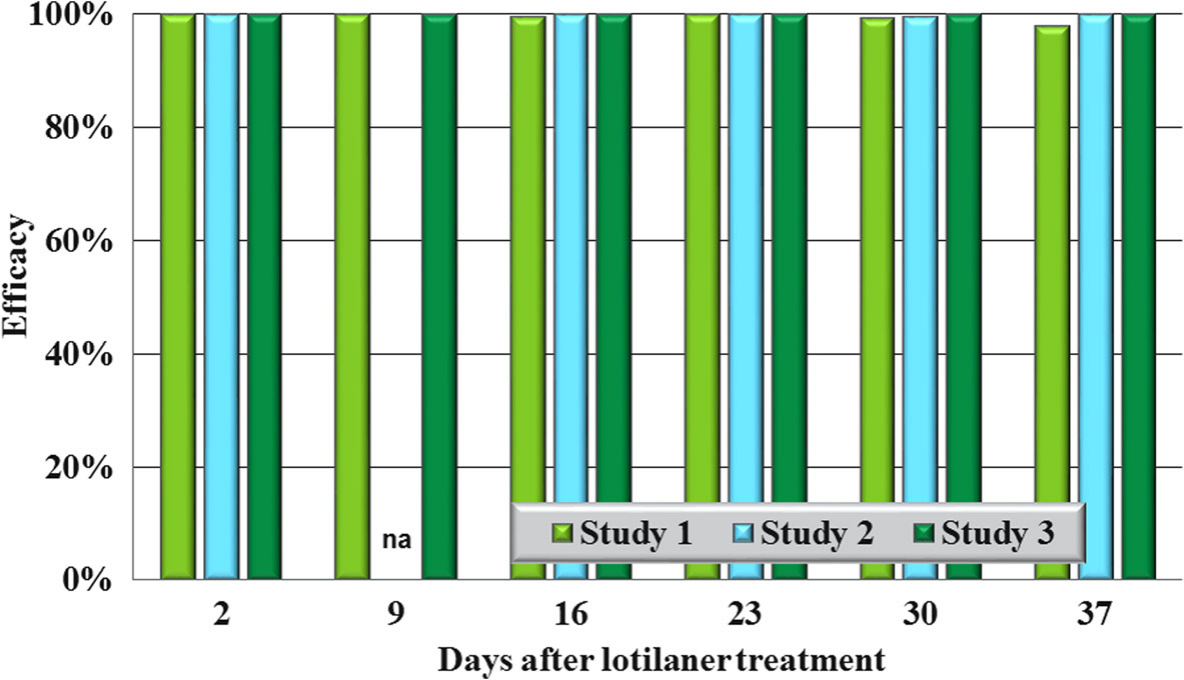
Percent reduction in geometric mean tick counts in lotilaner-treated dogs compared to untreated control dogs on each count day in each study for *Dermacentor variabilis*. *Abbreviation*: na, not applicable because of insufficient infestations in control dogs

**Fig. 2:**
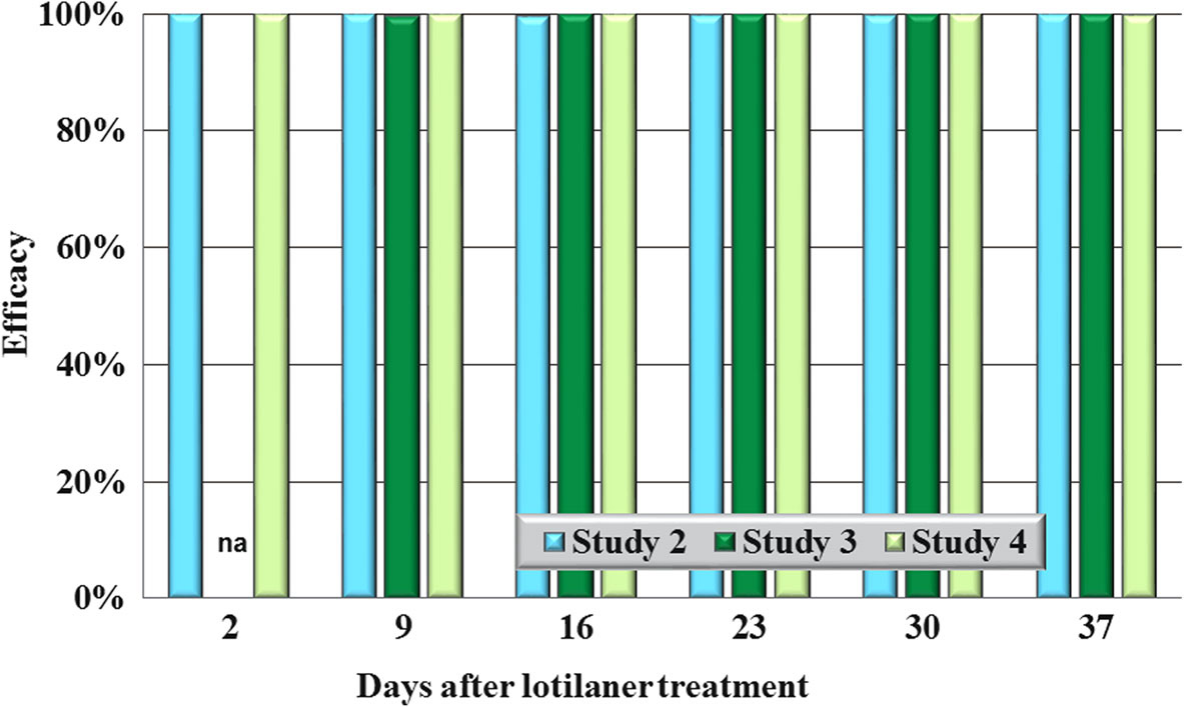
Percent reduction in geometric mean tick counts in lotilaner-treated dogs compared to untreated control dogs on each count day in each study for *Rhipicephalus sanguineus*. *Abbreviation*: na, not applicable because of insufficient infestations in control dogs

**Fig. 3:**
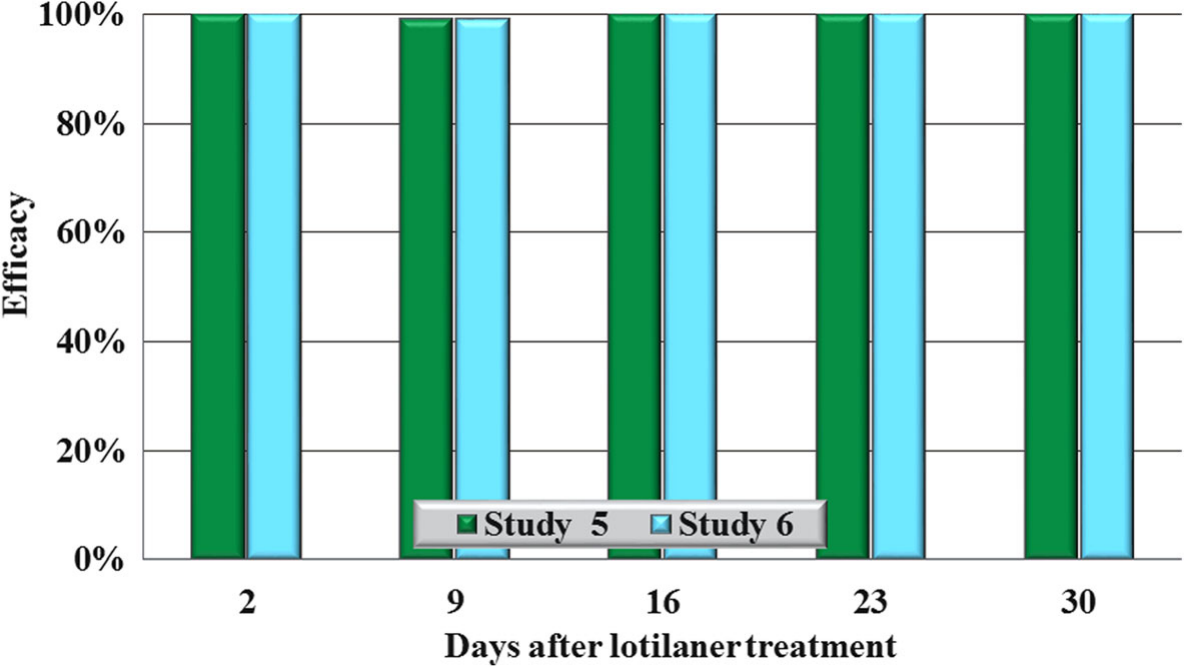
Percent reduction in geometric mean tick counts in lotilaner-treated dogs compared to untreated control dogs on each count day in each study for *Amblyomma americanum*

**Fig. 4:**
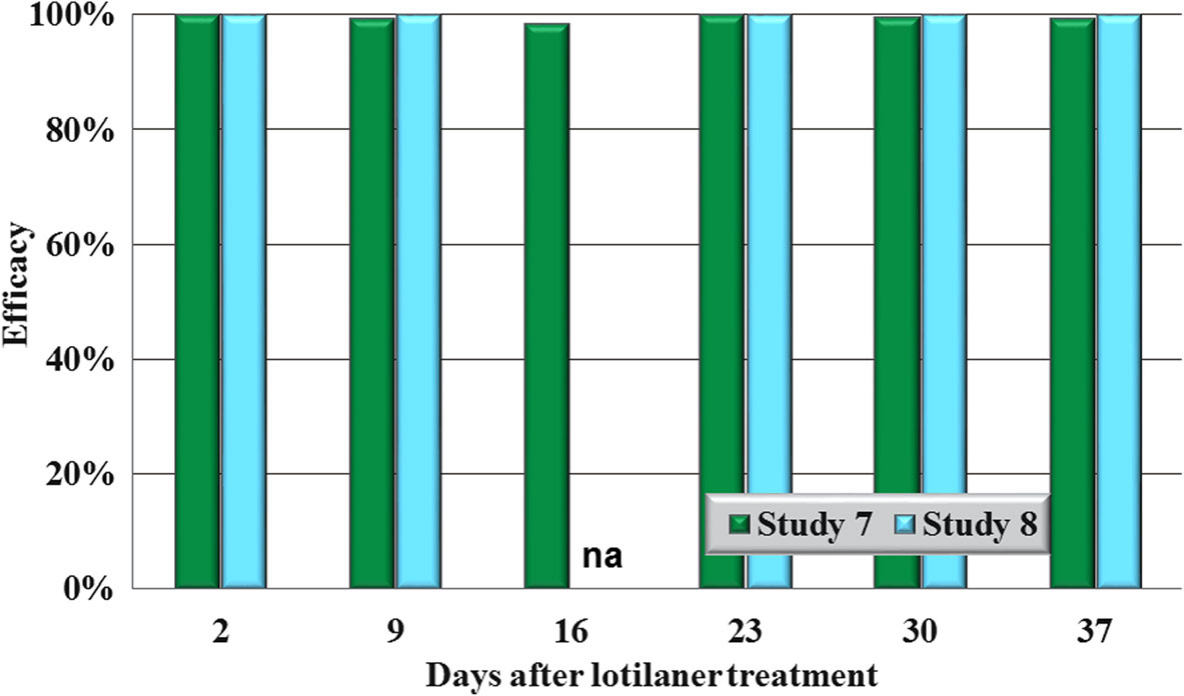
Percent reduction in geometric mean tick counts in lotilaner-treated dogs compared to untreated control dogs on each count day in each study for *Ixodes scapularis*. *Abbreviation*: na, not applicable because of insufficient infestations in control dogs

In all eight studies against the four species tested, on Day 2 (48 h post-treatment), no live ticks were found on any lotilaner-treated dog (Tables 1, 2, 3, 4; Figs. 1, 2, 3, 4). The high efficacy against ticks was sustained in all studies throughout the post-treatment assessment periods, and on only two occasions did geometric mean live tick count efficacy fall below 99%: in a *D. variabilis* study this was due to the presence on Day 35 of three live unengorged ticks on a single dog, and in an *I. scapularis* study on Day 16 a single dog had three live attached, engorged ticks and one dog had one live attached, engorged tick.

**Table 1:** Geometric (arithmetic) mean counts of live *Dermacentor variabilis* ticks

	Day	Untreated control		Lotilaner			Comparison
		Mean	Range	Mean	Range	Geometric (arithmetic) mean efficacy (%)	
Study 1	2	33.6 (35.0)	22–48	0.0 (0.0)	0–0	100 (100)	*t*_(7)_ = 32.0, *P* < 0.0001
	9	20.8 (23.0)	11–42	0.0 (0.0)	0–0	100 (100)	*t*_(7)_ = 18.7, *P* < 0.0001
	16	20.0 (24.0)	5–44	0.1 (0.1)	0–1	99.5 (99.5)	*t*_(7)_ = 11.8, *P* < 0.0001
	23	21.5 (24.3)	9–46	0.0 (0.0)	0–0	100 (100)	*t*_(7)_ = 16.7, *P* < 0.0001
	30	12.7 (18.3)	0–38	0.1 (0.1)	0–1	99.3 (99.3)	*t*_(7)_ = 6.2, *P* = 0.0004
	37	14.5 (19.6)	2–42	0.3 (0.5)	0–3	98.0 (97.5)	*t*_(7)_ = 7.2, *P* = 0.0002
Study 2	2	24.7 (25.0)	18–30	0.0 (0.0)	0–0	100 (100)	*t*_(14)_ = 53.5, *P* < 0.0001
	9	7.1 (9.0)	3–23	0.0 (0.0)	0–0	na	na
	16	20.3 (21.9)	10–35	0.0 (0.0)	0–0	100 (100)	*t*_(13)_ = 24.0, *P* < 0.0001
	23	29.4 (30.1)	22–41	0.0 (0.0)	0–0	100 (100)	*t*_(13)_ = 42.5, *P* < 0.0001
	30	21.9 (22.3)	15–28	0.1 (0.1)	0–1	99.6 (99.4)	*t*_(14)_ = 27.6, *P* < 0.0001
	37	29.6 (30.5)	17–40	0.0 (0.0)	0–0	100 (100)	*t*_(13)_ = 39.5, *P* < 0.0001
Study 3	2	16.6 (17.6)	11–36	0.0 (0.0)	0–0	100 (100)	*t*_(7)_ = 23.6, *P* < 0.0001
	9	14.2 (20.6)	0–41	0.0 (0.0)	0–0	100 (100)	*t*_(7)_ = 6.5, *P* = 0.0003
	16	21.3 (22.1)	13–33	0.0 (0.0)	0–0	100 (100)	*t*_(7)_ = 30.8, *P* < 0.0001
	23	26.8 (27.8)	18–41	0.0 (0.0)	0–0	100 (100)	*t*_(7)_ = 34.5, *P* < 0.0001
	30	31.8 (32.1)	25–39	0.0 (0.0)	0–0	100 (100)	*t*_(7)_ = 70.1, *P* < 0.0001
	37	19.2 (21.3)	9–36	0.0 (0.0)	0–0	100 (100)	*t*_(7)_ = 17.7, *P* < 0.0001

*Abbreviation*: *na* not applicable because of insufficient infestations in control dogs

**Table 2:** Geometric (arithmetic) mean counts of live *Rhipicephalus sanguineus* ticks

	Day	Untreated control		Lotilaner			Comparison
		Mean	Range	Mean	Range	Geometric (arithmetic) mean efficacy (%)	
Study 2	2	25.5 (27.6)	7–36	0.0 (0.0)	0–0	100 (100)	*t*_(13)_ = 19.1, *P* < 0.0001
	9	25.1 (26.0)	15–40	0.0 (0.0)	0–0	100 (100)	*t*_(14)_ = 32.0, *P* < 0.0001
	16	22.9 (24.5)	9–36	0.1 (0.3)	0–2	99.4 (99.0)	*t*_(14)_ = 15.1, *P* < 0.0001
	23	28.6 (29.9)	19–47	0.1 (0.1)	0–1	99.7 (99.6)	*t*_(14)_ = 23.9, *P* < 0.0001
	30	21.9 (23.4)	10–35	0.1 (0.1)	0–1	99.6 (99.5)	*t*_(14)_ = 18.7, *P* < 0.0001
	37	26.1 (28.1)	13–45	0.0 (0.0)	0–0	100 (100)	*t*_(13)_ = 23.3, *P* < 0.0001
Study 3	2	5.2 (6.5)	2–20	0.0 (0.0)	0–0	na	na
	9	14.3 (16.0)	4–27	0.1 (0.1)	0–1	99.4 (99.2)	*t*_(7)_ = 12.7, *P* < 0.0001
	16	29.5 (31.0)	14–43	0.0 (0.0)	0–0	100 (100)	*t*_(7)_ = 27.8, *P* < 0.0001
	23	26.5 (27.8)	17–42	0.0 (0.0)	0–0	100 (100)	*t*_(7)_ = 29.2, *P* < 0.0001
	30	19.6 (20.4)	12–31	0.0 (0.0)	0–0	100 (100)	*t*_(7)_ = 28.4, *P* < 0.0001
	37	14.8 (15.3)	10–25	0.0 (0.0)	0–0	100 (100)	*t*_(7)_ = 31.5, *P* < 0.0001
Study 4	2	34.2 (35.4)	17–45	0.0 (0.0)	0–0	100 (100)	*t*_(4)_ = 37, *P* < 0.0001
	9	33.6 (34.4)	24–43	0.0 (0.0)	0–0	100 (100)	*t*_(4)_ = 49.6, *P* < 0.0001
	16	30.7 (31.4)	19–42	0.0 (0.0)	0–0	100 (100)	*t*_(4)_ = 42.8, *P* < 0.0001
	23	29.9 (30.5)	20–37	0.0 (0.0)	0–0	100 (100)	*t*_(4)_ = 51.9, *P* < 0.0001
	30	28.4 (29.5)	16–39	0.0 (0.0)	0–0	100 (100)	*t*_(4)_ = 36.2, *P* < 0.0001
	37	27.2 (28.3)	15–37	0.1 (0.1)	0–1	99.7 (99.6)	*t*_(4)_ = 33.5, *P* < 0.0001

*Abbreviation*: *na* not applicable because of insufficient infestations in control dogs

**Table 3:** Geometric (arithmetic) mean counts of live *Amblyomma americanum* ticks

	Day	Untreated control		Lotilaner			Comparison
		Mean	Range	Mean	Range	Geometric (arithmetic) mean efficacy (%)	
Study 5	2	18.1 (18.8)	12–28	0.0 (0.0)	0–0	100 (100)	*t*_(7)_ = 29.3, *P* < 0.0001
	9	16.5 (17.8)	9–29	0.1 (0.1)	0–2	99.1 (98.6)	*t*_(7)_ = 18.4, *P* < 0.0001
	16	18.0 (18.6)	12–24	0.0 (0.0)	0–0	100 (100)	*t*_(7)_ = 29.1, *P* < 0.0001
	23	16.0 (16.4)	12–23	0.0 (0.0)	0–0	100 (100)	*t*_(7)_ = 35.6, *P* < 0.0001
	30	16.3 (16.5)	12–20	0.0 (0.0)	0–0	100 (100)	*t*_(7)_ = 53.3, *P* < 0.0001
Study 6	2	19.4 (20.0)	14–34	0.0 (0.0)	0–0	100 (100)	*t*_(9)_ = 38.8, *P* < 0.0001
	9	16.2 (17.5)	5–27	0.1 (0.1)	0–1	99.0 (98.9)	*t*_(9)_ = 18.3, *P* < 0.0001
	16	16.9 (18.8)	5–36	0.0 (0.0)	0–0	100 (100)	*t*_(9)_ = 18.0, *P* < 0.0001
	23	18.1 (18.7)	10–24	0.0 (0.0)	0–0	100 (100)	*t*_(9)_ = 35.0, *P* < 0.0001
	30	18.3 (19.3)	8–27	0.0 (0.0)	0–0	100 (100)	*t*_(9)_ = 26.6, *P* < 0.0001

**Table 4:** Geometric (arithmetic) mean counts of live *Ixodes scapularis* ticks

	Day	Untreated control		Lotilaner			Comparison
		Mean	Range	Mean	Range	Geometric (arithmetic) mean efficacy (%)	
Study 7	2	26.3 (27.5)	20–44	0.0 (0.0)	0–0	100 (100)	*t*_(6)_ = 24.7, *P* < 0.0001
	9	13.8 (15.1)	4–23	0.1 (0.1)	0–1	99.3 (99.2)	*t*_(14)_ = 13.4, *P* < 0.000
	16	18.9 (19.6)	12–27	0.3 (0.5)	0–3	98.4 (97.5)	*t*_(6)_ = 9.8, *P* < 0.0001
	23	27.1 (27.8)	20–38	0.0 (0.0)	0–0	100 (100)	*t*_(6)_ = 37.0, *P* < 0.0001
	30	21.0 (21.3)	17–28	0.1 (0.1)	0–1	99.6 (99.4)	*t*_(14)_ = 28.6, *P* < 0.0001
	37	27.0 (28.0)	14–38	0.2 (0.3)	0–1	99.3 (99.1)	*t*_(6)_ = 18.5, *P* < 0.0001
Study 8	2	18.4 (18.6)	14–24	0.0 (0.0)	0–0	100 (100)	*t*_(7)_ = 51.0, *P* < 0.0001
	9	14.6 (15.0)	9–21	0.0 (0.0)	0–0	100 (100)	*t*_(7)_ = 30.4, *P* < 0.0001
	16	11.8 (12.8)	7–21	0.0 (0.0)	0–0	na	na
	23	13.6 (13.9)	10–19	0.0 (0.0)	0–0	100 (100)	*t*_(7)_ = 37.9, *P* < 0.0001
	30	10.6 (13.8)	0–20	0.0 (0.0)	0–0	100 (100)	*t*_(7)_ = 6.9, *P* = 0.0002
	37	14.1 (14.3)	11–17	0.0 (0.0)	0–0	100 (100)	*t*_(7)_ = 55.1, *P* < 0.0001

*Abbreviation*: *na* not applicable because of insufficient infestations in control dogs

There were no serious adverse events in lotilaner-treated dogs, and the observations of transient non-serious events were consistent with those expected to occur in laboratory dogs, particularly those exposed to ticks, irrespective of treatment. These events, which occurred in treated and control groups, included mild skin disorder of crusts and focal alopecia observed at sites of tick attachment, with lower frequency in the lotilaner-treated dogs. There were isolated incidents of loose stool, and one lotilaner-treated dog vomited following sedation with medetomidine. None of the adverse events was regarded as being related to administration of lotilaner, and none required treatment.

## Discussion

In each study, lotilaner had eliminated all infesting ticks by 48 h post-treatment. Against new infestation challenges post-treatment, a single dose of lotilaner provided consistent and sustained activity. At the Day 30 counts, lotilaner was 100% effective against *A. americanum*, and efficacy of at least 98.0% was maintained against *D. variabilis*, *R. sanguineus* and *I scapularis*. For these three species, efficacy against a Day 35 challenge was 98% to 100%, showing that lotilaner sustains its efficacy through the end of the 1 month period and that there is no end-of-dose decline in efficacy.

The results reported herein compare favourably with earlier isoxazoline reports that assessed the use of isoxazolines against ticks. For instance, lotilaner efficacy was 100% against *R. sanguineus* and *I. scapularis* in at least 50% of post-treatment challenges. Another isoxazoline, sarolaner achieved similar results against these tick species, based on 48-h counts, through at least 1 month after treatment [13]. In contrast, afoxolaner, also an isoxazoline, did not achieve 100% efficacy 48 h after any post-treatment challenge in two studies with *R. sanguineus* [14]*.* Similarly with *I. scapularis*, according to the product’s Freedom of Information summary, two studies of afoxolaner-treated dogs indicated that efficacy did not reach 100% against post-treatment challenges, although a published report of one of these studies described 100% efficacy against the challenge at Day 7 [15, 16].

The effectiveness of lotilaner demonstrated in these eight studies indicates its strong potential for use in the treatment and control of canine tick infestations. This may be particularly important in geographies where there needs to be heightened confidence in the sustained end-of-dose effectiveness of a tick control product. Results of other studies have indicated that lotilaner has a rapid onset (within 4 h) of activity in dogs after treatment both against fleas and against the tick *Ixodes ricinus* [8, 9, 17]. Thus with a rapid onset of activity and sustained action through the month following treatment, lotilaner can be a valuable safe and effective addition to a veterinarian and a pet owner’s armamentarium for the control of fleas and ticks. The high efficacy maintained at Day 37 against *D. variabilis*, *R. sanguineus* and *I. scapularis* and the almost 100% efficacy from Day 0 to Day 30 against *A. americanum* also provide comfort to veterinarians and owners that there will be minimal risk of infestations if a scheduled monthly dosing is delayed by a few days.

## Conclusion

Lotilaner provided excellent efficacy against existing infestations with the common species of ticks that affect dogs in North America, with a sustained efficacy of at least 98% for at least 4 weeks against subsequent challenge infestations. These results provide assurance that lotilaner is a highly effective isoxazoline that offers sustained efficacy against ticks through and beyond the one-month end-of-dose treatment interval.

## Additional file

Additional file 1: Spanish translation of the article. (PDF 147 kb)

## Abbreviations

GLP: Good laboratory practice
Mc: Mean number of live ticks (per species on animals) in the untreated control group
Mt: Mean number of live ticks on animals in the treated group
Na: Not applicable because of insufficient infestations in control dogs
