# Supplementary material for: Laboratory evaluations of the immediate and sustained efficacy of lotilaner (Credelio™) against four common species of ticks affecting dogs in North America
Source: Parasit Vectors. 2017 Nov 1;10:523. doi: 10.1186/s13071-017-2476-y (PMC5664823; doi:10.1186/s13071-017-2476-y)
Supplement: Additional file 1: — Spanish translation of the article. (PDF 147 kb) [file 13071_2017_2476_MOESM1_ESM.pdf]

Evaluaciones de laboratorio de la eficacia inmediata y sostenida de lotilaner (Credelio™) contra cuatro especies comunes de garrapatas que afectan a los perros en Norte América

Martin Murphy[1], Roberto Garcia[2], Daniela Karadzovska[3], Daniela Cavalleri[1], Dan Snyder[2], Wolfgang Seewald[1], Theresa Real[2], Jason Drake[2]*, Scott Wiseman[4] y Steve Nanchen[1]

[1]Elanco Animal Health, Schwarzwaldallee 215, CH-4058 Basel WRO-1032.2.58, Suiza
[2]Elanco Animal Health, 2500 Innovation Way, Greenfield, IN 46140, E.U.A.
[3]Elanco Animal Health, Yarrandoo, New South Wales, Australia
[4]Elanco Animal Health, Southampton, Hants, RU

*Correspondencia: drake_jon_j@elanco.com

Martin Murphy: murphy_martin_gerard@elanco.com; Roberto Garcia: roberto-3.garcia@elanco.com; Daniela Karadzovska: daniela.karadzovska@elanco.com; Daniela Cavalleri: cavalleri_daniela_a@elanco.com; Daniel E. Snyder: snyder_daniel_e@elanco.com; Wolfgang Seewald: seewald_wolfgang@elanco.com; Theresa Real: real.theresa@elanco.com; Jason Drake: drake_jon_j@elanco.com; Scott Wiseman: wiseman_scott@elanco.com; Steve Nanchen: nanchen_steve@elanco.com

Resumen

Antecedentes: El control efectivo de las infestaciones de garrapatas en perros es importante para reducir el riesgo de la transmisión de patógenos bacterianos, virales y de protozoarios. Se iniciaron estudios de laboratorio para determinar la eficacia de lotilaner contra las garrapatas comunes que infestan perros en los Estados Unidos.

Métodos: En ocho estudios se investigó la eficacia de lotilaner contra garrapatas. En dos estudios se infestaron perros con *Dermacentor variabilis* y *Rhipicephalus sanguineus*: un estudio adicional se completó para cada una de estas especies. Dos estudios evaluaron infestaciones con *Amblyomma americanum* y dos con *Ixodes scapularis*. En todos los estudios, los perros se clasificaron y se bloquearon por conteos de infestaciones pre-tratamiento y se ubicaron al azar, al menos ocho por

grupo, para ser tratados oralmente con lotilaner (nivel mínimo de dosis de 20 mg/kg), o para ser controles no tratados. Los tratamientos se administraron en el Día 0, al cabo de 30 minutos después los perros fueron alimentados. En todos los estudios, se llevaron a cabo infestaciones con 50 garrapatas adultas en los Días -2, 7, 14, 21, y 28, y también en el Día 35 para *R. sanguineus, D. variabilis* e *I. scapularis.* Los conteos de garrapatas se completaron a las 48 horas después del tratamiento o después de cada desafío subsecuente. Se definió una infestación adecuada como al menos 25% de la dosis de infestación recuperada de cada uno de al menos seis animales control en cada evaluación. Los cálculos de eficacia para el objetivo primario se basaron en las medias geométricas.

Resultados: En todos los estudios, lotilaner fue 100% efectivo contra las infestaciones existentes. Para las evaluaciones post-tratamiento, en solo dos ocasiones la eficacia cayó por debajo del 99%: en un estudio con *D. variabilis* la eficacia fue del 98.0% en el Día 35 y en un estudio con *I. scapularis* la eficacia en el Día 16 fue del 98.4%. Solamente se observaron eventos adversos leves y transitorios, y ninguno fue considerado que se relacionara con el tratamiento.

Conclusión: Lotilaner fue completamente efectivo contra las infestaciones existentes con cuatro especies comunes de garrapatas, *D. variabilis, R. sanguineus, A. americanum* e *I. scapularis,* que afectan perros en Norte América, con al menos cuatro semanas de eficacia del 98.0% o más contra las infestaciones de desafío subsecuentes. Estos resultados demuestran que lotilaner es una isoxazolina altamente efectiva que ofrece eficacia sostenida contra garrapatas a lo largo y más allá del mes de intervalo de tratamiento al final-de-la-dosis.

Palabras clave: Lotilaner, Credelio, Garrapatas, *Ixodes*, *Rhipicephalus*, *Amblyomma*, *Dermacentor*, Perro

Antecedentes

El control efectivo de las infestaciones por garrapata en perros es importante para reducir el riesgo de la transmisión de una gran variedad de patógenos bacterianos, virales y de protozoarios. Las garrapatas también son una fuente de irritación en el sitio de adhesión, y cuando se adhieren al hospedador puede ser difícil removerlas. Una remoción inadecuada puede llevar a consecuencias inflamatorias a largo plazo [1]. Por lo tanto, el control de la garrapata durante todo el año es importante, y los productos que se utilizan deberán de ser efectivos contra las garrapatas adheridas y proveer protección post-tratamiento que limite el riesgo de una adhesión prolongada de nuevos

desafíos, particularmente porque los niveles acaricidas pueden disminuir hacia el final del período entre-dosis (final-de-la-dosis).

Hay cuatro especies de garrapatas que comúnmente infestan a los perros en Norte América que son *Dermacentor variabilis, Rhipicephalus sanguineus*, *Amblyomma americanum* e *Ixodes scapularis.* Hasta recientemente, el control de estas especies ha dependido de químicos aplicados tópicamente o el uso de collares que liberan a los químicos sobre el pelaje del animal tratado. Ambas medidas implican un riesgo para el propietario por la exposición al plaguicida, o la ingestión oral por las mascotas tratadas o en-contacto [2]. Una limitante de productos aplicados externamente es el potencial para los factores climáticos y la exposición al agua para reducir la eficacia continua [3]. Adicionalmente, para asegurar la efectividad, los productos tópicos deben de ser aplicados cuidadosamente por el propietario.

El descubrimiento de la nueva familia de compuestos, las isoxazolinas, ha permitido desarrollar productos de administración oral con el potencial para proveer la eliminación de pulgas y garrapatas que están infestando y la actividad contra el desafío post-tratamiento [4-6]. Lotilaner es una isoxazolina recientemente desarrollada que se ha demostrado que tiene una rápida absorción y una vida media prolongada después de la administración oral de una tableta masticable saborizada para perros [7]. Se ha visto que esta rápida absorción de lotilaner se ha traducido en un rápido inicio de la actividad contra pulgas con una alta eficacia sostenida hasta los 35 días después de un solo tratamiento [8, 9]. Para determinar si lotilaner proporcionaría una eficacia sostenida equivalente contra las garrapatas, se llevaron a cabo ocho estudios en los que los perros tratados se desafiaron con una o más de cada una de las cuatro especies de garrapatas más comunes que infestan perros en Norte América.

El objetivo de cada estudio fue determinar la eficacia y la seguridad de las tabletas masticables saborizadas de lotilaner cuando se dosificaron perros oralmente a una dosis mínima de 20 mg/kg contra infestaciones de garrapatas presentes al momento de que se administró el tratamiento, o contra nuevas infestaciones a los 28 a 35 días post-tratamiento. En dos estudios los perros se infestaron con *D. variabilis* y *R. sanguineus* (Estudios 1 y 2) y hubo un estudio adicional para cada una de esas especies (Estudios 3 y 4, respectivamente). Se condujeron estudios para *A. americanum* (Estudios 5 y 6) e *I. scapularis* (Estudios 7 y 8). En los estudios de *A. americanum*, muy pocas garrapatas estaban disponibles para permitir los desafíos significativos más allá del Día 28. Todos los estudios fueron en un solo centro, el investigador/asesor – ciego, y aleatorizado con ocho o diez perros sanos por grupo de estudio.

Métodos

Los estudios se completaron en los laboratorios en los Estados Unidos (Arkansas, Georgia, y Texas), Irlanda y Suiza. Se prepararon los protocolos en consideración de las recomendaciones establecidas en las guías de la Asociación Mundial para los Avances de Parasitología Veterinaria para la evaluación de la eficacia de parasiticidas para el tratamiento, prevención y control de las infestaciones de pulgas y garrapatas en perros y gatos [10]. Los estudios se condujeron de acuerdo a los principios de Buenas Prácticas Clínicas y Buenas Prácticas de Laboratorio (GLP) para Estudios de Laboratorio No-Clínico [11, 12].

Animales y alojamiento

En siete de los ocho estudios se enrolaron 16 perros Beagle; 20 perros se enrolaron en el Estudio 6. Los perros tenían al menos seis meses de edad y pesaban entre 6.7 a 20.0 kg. Antes de enrolarlos, todos los perros habían estado aclimatizados a las instalaciones del estudio. Para calificar para la inclusión se requería que cada perro estuviera sano y demostrara la susceptibilidad a la infestación por garrapatas basado en la retención de al menos un 25% del desafío de garrapata conducido, dependiendo del estudio, del Día -14 al Día -7. Se excluyeron los perros si estaban gestantes o lactando, o sí habían sido expuestos a tratamientos ectoparasiticidas por ocho semanas a seis meses antes del enrolamiento en el estudio, dependiendo de la duración de la actividad del producto utilizado. Todos los perros se alojaron individualmente durante los períodos de desafío con las garrapatas, tuvieron acceso a agua *ad libitum* y se alimentaron con una dieta canina de alta calidad comercialmente disponible de acuerdo al procedimiento estándar de cada instalación.

Infestaciones con garrapatas y conteos

Las infestaciones se completaron por medio de una aplicación gentil en la grupa dorsal o lateral o el abdomen del perro, ya sea con sedación con medemotidina, o contención manual sin sedación por hasta un minuto para permitir que las garrapatas se desplazaran entre el pelo del perro. Para cada estudio, las infestaciones se completaron aplicando aproximadamente 50 garrapatas de las especies relevantes (aproximadamente proporción 1:1, macho:hembra) en el Día -2, y post-tratamiento en los Días 7, 14, 21, y 28, y también en el Día 35 para *R. sanguineus*, *D. variabilis* e *I. scapularis*.

Las garrapatas de cada especie probada se obtuvieron de las colonias mantenidas en el laboratorio. Todas las colonias se iniciaron con aislamientos de campo y se habían refrescado en

intervalos anuales o bianuales con garrapatas adicionales capturadas en el campo de diferentes áreas. En un estudio de *I. scapularis*, las garrapatas se colectaron en el campo por medio de marcaje de la vegetación en Carolina del Sur. Las garrapatas capturadas se mantuvieron en viales de vidrio con papel filtro humedecido y se colocaron en una cámara que proporcionó una humedad relativa y temperatura de aproximadamente 98.5% y 11.1 °C, respectivamente.

Las remociones de los perros y el conteo de las garrapatas vivas se completaron en aproximadamente 48 horas post-tratamiento y aproximadamente 48 horas después de cada infestación subsecuente. El procedimiento del conteo de garrapatas para el estudio consistió en un examen complete de todas las áreas del cuerpo por palpación con las puntas de los dedos (conteo con el pulgar) primero localizando y contando las garrapatas adheridas/libres seguido del peinado del pelaje del animal para remover todas las garrapatas. Las garrapatas se consideraron vivas si las patas reaccionaban a un estímulo táctil o a exhalar aire (bióxido de carbono) y se consideraban muertas sí no lo hacían.

Aleatorización y tratamiento

Ente una y dos semanas antes de la administración del tratamiento del estudio, una infestación pre-tratamiento se llevó a cabo y las garrapatas se removieron y se contaron 48 horas después. Se usaron los conteos de garrapatas vivas, adheridas para su clasificación, aleatorización y formación de bloques de cada perro a uno de los dos grupos de tratamiento.

En cada estudio, los perros en un grupo recibieron tabletas masticables saborizadas de lotilaner, administradas oralmente tan cerca como fuera posible al nivel de dosis mínima recomendada de 20 mg/kg, sin subdosificar. Basándose en los tamaños disponibles de las tabletas de 56.25 mg, 112.5 mg y 225 mg se dosificaron los perros con la mejor combinación de tabletas para alcanzar su nivel de dosis objetivo, de acuerdo con su peso corporal. Los perros en el otro grupo no se trataron o se trataron con un tratamiento inefectivo como controles negativos para facilitar que el personal del estudio estuviera ciego. Todos los perros consumieron al menos un tercio de su ración diaria al cabo de 30 minutos antes de dosificarlos. En el Día 0, los tratamientos se administraron directamente en la boca de cada perro para asegurar que la dosis objetivo se estaba administrando.

Las observaciones clínicas se hicieron antes de dosificar y a aproximadamente una, seis y ocho horas (± 15 minutos) post-dosis para observar eventos adversos. Las observaciones post-dosis de la salud de cada perro se llevaron a cabo al menos una vez al día hasta el final del estudio.

Evaluación de la eficacia

El perro individual fue la unidad experimental. La eficacia de lotilaner se calculó por separado para cada especie de garrapata por medio de la comparación de las medias geométricas y aritméticas de los conteos en el grupo de lotilaner contra aquellos en el grupo control no tratado. La fórmula de Abbott se utilizó para calcular la eficacia.

$$\text{Porcentaje de eficacia} = 100 \times (Mc - Mt)/Mc$$

en dónde, Mc es la media del número de garrapatas vivas (por especie en los animales) en el grupo control no tratado, y Mt es la media del número de garrapatas vivas en los animales en el grupo tratado.

Hubo cálculos separados para cada especie de garrapata en cada conteo en un punto en el tiempo. Puesto que el cálculo de la media geométrica involucró tomar el logaritmo del conteo de garrapatas para cada animal, para cualquier conteo de garrapatas que fuera igual de cero a uno (1) se agregaba al conteo para cada animal en cada grupo. Sí se agregaba un uno (1) al conteo de garrapatas, esta constante (1) se restaba de la media geométrica calculada resultante antes de calcular la eficacia porcentual.

Lotilaner se consideró que era efectivo en un punto de tiempo dado si el siguiente criterio se cumplía para el grupo de tratamiento y las especies de garrapatas en ese punto en el tiempo: (i) Una infestación adecuada se logró en los perros del grupo control. Una infestación adecuada se definió como al menos un 25% de la dosis de infestación (i.e. ≥ 12 garrapatas) recuperada de cada uno de al menos 6 animales control en cada evaluación; (ii) hubo una diferencia estadística significativa (nivel de dos-lados, $P < 0.05$) en la media geométrica de los conteos de garrapatas entre el grupo tratado y el grupo control no tratado, con un número significativamente más bajo del número de garrapatas vivas (adheridas y no adheridas) en el grupo tratado en comparación con el control negativo; (iii) el grupo tratado tuvo una eficacia calculada de la menos un 90%.

Resultados

Se demostró la infestación adecuada (al menos seis perros control no tratados con una tasa de adherencia mayor que el 25% para las especies de garrapatas probadas, y una tasa de infestación del grupo control promedio mayor al 25%, o 12 garrapatas) en todos menos tres estudios. Las

excepciones fueron las tasas de infestación en el grupo control en un estudio de *D. variabilis* (Estudio 2, en el Día 9), en un estudio de *R. sanguineus* (Estudio 3, Día 2), y en un estudio con *I. scapularis* colectadas en el campo (Estudio 8, Día 16). En cada una de estas evaluaciones, no hubo garrapatas vivas en los perros tratados con lotilaner.

En los ocho estudios contra las cuatro especies tratadas, en el Día 2 (48 horas post-tratamiento), no se encontraron garrapatas vivas en ningún perro tratado con lotilaner (Tablas 1–4; Figs. 1–4). La elevada eficacia contra garrapatas fue sostenida en todos los estudios a lo largo de los períodos de evaluación post-tratamiento, y en solo dos ocasiones la media geométrica de la eficacia del conteo de garrapatas vivas cayó abajo del 99%: en un estudio con *D. variabilis* se debió a la presencia de tres garrapatas ingurgitadas vivas en el Día 35 en un solo perro, y en un estudio de *I. scapularis* en el Día 16 un solo perro tuvo tres garrapatas ingurgitadas, vivas y un perro tuvo una garrapata ingurgitada, adherida, viva.

No hubieron eventos adversos severos en los perros tratados con lotilaner, y las observaciones de eventos transitorios no severos fueron consistentes con aquellos que se esperaban presentar en los perros de laboratorio, particularmente aquellos expuestos a las garrapatas, independientemente del tratamiento. Estos eventos, que ocurrieron en los grupos tratados y control, incluyeron un desorden leve en la piel con costras y alopecia focal observada en los sitos de adhesión de las garrapatas, con menor frecuencia en los perros tratados con lotilaner. Hubo incidentes aislados de heces sueltas, y un perro tratado con lotilaner vomitó después de la sedación con medetomidina. Ninguno de los eventos adversos fue considerado que tuviera relación con la administración de lotilaner, y ninguno requirió tratamiento.

Discusión

En cada estudio, lotilaner eliminó a las garrapatas infestantes alas 48 horas post-tratamiento. Contra los nuevos desafíos de infestación post-tratamiento, una sola dosis de lotilaner proporcionó actividad consistente y sostenida. En los conteos del Día 30, lotilaner fue efectivo al 100% contra *A. americanum*, y una eficacia de al menos 98.0% se mantuvo contra *D. variabilis, R. sanguineus* e *I scapularis*. Para estas tres especies, la eficacia contra el desafío del Día 35 fue del 98% al 100%, mostrando que lotilaner sostiene su eficacia hasta el final de un período de un mes y no hubo una disminución en la misma al-final-de-la-dosis.

Los resultados reportados de aquí en adelante se comparan favorablemente con los primeros reportes de isoxazolina que evaluaron el uso de las isoxazolinas contra garrapatas. Por ejemplo, la eficacia de lotilaner fue del 100% contra *R. sanguineus* e *I. scapularis* en al menos un 50% de los desafíos post-tratamiento. Otra isoxazolina, el sarolaner logró resultados similares contra estas especies de garrapata, basado en los conteos de 48-horas, a lo largo de al menos un mes después del tratamiento [13]. En contraste, afoxolaner, también una isoxazolina, no logró el 100% de eficacia en 48 horas después de cualquier desafío post-tratamiento en dos estudios con *R. sanguineus* [14]. De manera similar, con *I. scapularis*, de acuerdo con el resumen de Libertad de Información de un producto, dos estudios en perros tratados con afoxolaner indicaron que la eficacia no alcanzó el 100% contra los desafíos post-tratamiento, aunque un reporte publicado de uno de estos estudios describió un 100% de eficacia contra el desafío en el Día 7 [15, 16].

La efectividad de lotilaner demostrada en estos ocho estudios indica su fuerte potencial para usarse en el tratamiento y control de las infestaciones por garrapatas en caninos. Esto puede ser particularmente importante en geografías en donde las necesidades deben enaltecer la confianza en una efectividad sostenida al-final-de-la-dosis para un producto que controle a la garrapatas. Los resultados de otros estudios han indicado que lotilaner tiene un inicio rápido(al cabo de cuatro horas) de actividad en perros después del tratamiento tanto contra pulgas, como contra la garrapata *Ixodes ricinus* [8, 9, 17]. Por ende, con un rápido inicio de la actividad y una acción sostenida a lo largo de un mes después del tratamiento, lotilaner puede ser una adición valiosa, segura y efectiva para el armamento del veterinario y del propietario de la mascota para el control de pulgas y garrapatas. La elevada eficacia mantenida al Día 37 contra *D. variabilis*, *R. sanguineus* e *I. scapularis* y el casi 100% de eficacia del Día 0 al Día 30 contra *A. americanum* también provee confort para los veterinarios y propietarios de que habrá un riesgo mínimo de infestaciones sí la dosificación mensual programada se retrasa un par de días.

Conclusión

Lotilaner proporcionó una eficacia excelente contra las infestaciones existentes con las especies comunes de garrapatas que afectan a los perros en Norte América, con una eficacia sostenida de al menos 98% por al menos cuatro semanas contra las infestaciones de desafío subsecuentes. Estos resultados aseguran que lotilaner es una isoxazolina altamente efectiva que ofrece una eficacia

sostenida contra garrapatas a lo largo y más allá de un mes al final-de-la-dosis del intervalo de tratamiento.

Abreviaturas

GLP: buenas prácticas de laboratorio; Mc: media del número de garrapatas vivas (por especies en animales) en el grupo control no tratado; Mt: media del número de garrapatas vivas en animales en el grupo tratado; na, no aplicable debido a infestaciones insuficientes en los perros control.

Reconocimientos

Los autores quisieran agradecer al Dr Bill Ryan de Ryan Mitchell Associates LLC por su asistencia con el manuscrito.

Declaraciones

Declaración de ética y consentimiento para participar

Todos los estudios fueron aprobados por el Grupo de Bienestar Animal de Novartis Animal Health Global.

Consentimiento para publicación

No aplicable.

Disponibilidad de datos y material

Debido a la confidencialidad comercial de esta investigación, los datos no incluidos en el manuscrito solo pueden estar disponibles a los investigadores *bona fide* sujetos a un acuerdo de no-divulgación.

Intereses competentes

MM, RG, DK, DC, DS, WS, TR, JD, SW y SN son empleados de Elanco.

Fondos

Todos los estudios fueron patrocinados por Elanco.

Contribuciones de los autores

Todos los autores participaron en el diseño, completaron los estudios y estuvieron involucrados en el manuscrito preliminar. Todos los autores leyeron y aprobaron el manuscrito final.

Detalles del autor

[1]Elanco Animal Health, Schwarzwaldallee 215, CH-4058 Basel WRO-1032.2.58, Suiza. [2]Elanco Animal Health, 2500 Innovation Way, Greenfield, IN 46140, E.U.A. [3]Elanco Animal Health, Yarrandoo, New South Wales, Australia. [4]Elanco Animal Health, Southampton, Hants, RU.

Referencias

1. Blagburn BL, Dryden MW. Biology, treatment, and control of flea and tick infestations. Vet Clin Small Anim. 2009;39:1173-1200.

2. Obendorf SK, Lemley AT, Hedge A, Kline AA, Tan K, Dokuchayeva T. Distribution of pesticide residues within homes in central New York State. Arch Environ Contam Toxicol. 2006;50:31-44.

3. Dryden MW, Ryan WG, Bell M, Rumschlag AJ, Young LM, Snyder DE. Assessment of owner-administered monthly treatments with oral spinosad or topical spot-on fipronil/(S)-methoprene in controlling fleas and associated pruritus in dogs. Vet Parasitol. 2013;191:340-6.

4. Shoop WL, Hartline EJ, Gould BR, Waddell ME, McDowell RG, Kinney JB, et al. Discovery and mode of action of afoxolaner, a new isoxazoline parasiticide for dogs. Vet Parasitol. 2014;201:179-89.

5. Williams H, Zoller H, Roepke RK, Zschiesche E, Heckeroth AR. Fluralaner activity against life stages of ticks using *Rhipicephalus sanguineus* and *Ornithodoros moubata* in in vitro contact and feeding assays. Parasit Vectors. 2015;8:90.

6. McTier TL, Chubb N, Curtis MP, Hedges L, Inskeep GA, Knauer CS, et al. Discovery of sarolaner: a novel, orally administered, broad-spectrum, isoxazoline ectoparasiticide for dogs. Vet Parasitol. 2016;222:3-11.

7. Toutain CE, Seewald W, Jung M. The intravenous and oral pharmacokinetics of lotilaner and the effect of food in dogs. Parasit Vectors, 2017 (In press).

8. Cavalleri D, Murphy M, Seewald W, Drake J, Nanchen S. Assessment of the onset of lotilaner (Credelio™) speed of kill of fleas on dogs. Parasit Vectors. 2017 (In press).

9. Cavalleri D, Murphy M, Seewald W, Drake J, Nanchen S. Assessment of the speed of flea kill of lotilaner (Credelio™) throughout the month following oral administration to dogs. Parasit Vectors. 2017 (In press).

10. Marchiondo AA, Holdsworth PA, Fourie LJ, Rugg D, Hellmann K, Snyder DE, et al. World Association for the Advancement of Veterinary Parasitology (W.A.A.V.P.) 2nd. ed.: Guidelines for

evaluating the efficacy of parasiticides for the treatment, prevention and control of flea and tick infestations on dogs and cats. Vet Parasitol. 2013;194:84–97.

11. European Medicines Agency, Committee for Medicinal Products for Veterinary Use, 2000. Guideline on Good Clinical Practices. VICH Topic GL9 http://www.ema.europa.eu/docs/en GB/document library/Scientific guideline/2009/10/WC500004343.pdf. Accessed January 11, 2017.

12. United States Food and Drug Administration. Good Laboratory Practices (GLP) for Non-Clinical Laboratory Studies. http://www.fda.gov/ohrms/dockets/98fr/980335s1.PDF Accessed January 16, 2017.

13. Six RH, Everett WR, Young DR, Carter L, Mahabir SP, Honsberger NA, et al. Efficacy of a novel oral formulation of sarolaner (Simparica™) against five common tick species infesting dogs in the United States. Vet Parasitol. 2016;222:28-32.

14. Kunkle B, Daly S, Dumont P, Drag M, Larsen D. Assessment of the efficacy of orally administered afoxolaner against *Rhipicephalus sanguineus sensu lato*. Vet Parasitol. 2014;201:226-8.

15. NexGard Freedom of Information Summary. http://www.fda.gov/downloads/AnimalVeterinary/Products/ApprovedAnimalDrugProducts/FOIADrugSummaries/UCM409102.pdf. Accessed December 14, 2016.

16. Mitchell EB, McCall JW, Theodore Chester S, Larsen D. Efficacy of afoxolaner against *Ixodes scapularis* ticks in dogs. Vet Parasitol. 2014;201:223-5.

17. Murphy M, Cavalleri D, Seewald W, Drake J, Nanchen S. Laboratory evaluation of the speed of kill of lotilaner (Credelio™) against *Ixodes ricinus* ticks on dogs. Parasit Vectors. (In press).

Leyendas de las figuras

Fig. 1 Porcentaje de reducción en la media geométrica de los conteos de garrapatas en los perros tratados con lotilaner comparados con perros control no tratados en cada día de conteo en cada estudio para *Dermacentor variabilis*.

Abreviatura: na, no aplicable debido a infestaciones insuficientes en los perros control

Fig. 2 Porcentaje de reducción en la media geométrica de los conteos de garrapatas en perros tratados con lotilaner comparados con perros control no tratados en cada día de conteo en cada estudio para *Rhipicephalus sanguineus*. *Abreviatura*: na, no aplicable debido a infestaciones insuficientes en los perros control

Fig. 3 Porcentaje de reducción en la media geométrica de los conteos de garrapatas en perros tratados con lotilaner comparados con perros control no tratados en cada día de conteo en cada estudio para *Amblyomma americanum*

Fig. 4 Porcentaje de reducción en la media geométrica de los conteos de garrapatas en perros tratados con lotilaner comparados con perros control no tratados en cada día de conteo en cada estudio para *Ixodes scapularis*.

Abreviatura: na, no aplicable debido a infestaciones insuficientes en los perros control

Tabla 1 Media geométrica (aritmética) de conteos de garrapatas vivas *Dermacentor variabilis*

	Día	Control no tratado		Lotilaner			Comparación
		Media	Rango	Media	Rango	Media geométrica (aritmética) de eficacia (%)	
Estudio 1	2	33.6 (35.0)	22–48	0.0 (0.0)	0–0	100 (100)	$t_{(7)} = 32.0$, $P < 0.0001$
	9	20.8 (23.0)	11–42	0.0 (0.0)	0–0	100 (100)	$t_{(7)} = 18.7$, $P < 0.0001$
	16	20.0 (24.0)	5–44	0.1 (0.1)	0–1	99.5 (99.5)	$t_{(7)} = 11.8$, $P < 0.0001$
	23	21.5 (24.3)	9–46	0.0 (0.0)	0–0	100 (100)	$t_{(7)} = 16.7$, $P < 0.0001$
	30	12.7 (18.3)	0–38	0.1 (0.1)	0–1	99.3 (99.3)	$t_{(7)} = 6.2$, $P = 0.0004$
	37	14.5 (19.6)	2–42	0.3 (0.5)	0–3	98.0 (97.5)	$t_{(7)} = 7.2$, $P = 0.0002$
Estudio 2	2	24.7 (25.0)	18–30	0.0 (0.0)	0–0	100 (100)	$t_{(14)} = 53.5$, $P < 0.0001$
	9	7.1 (9.0)	3–23	0.0 (0.0)	0–0	na	na
	16	20.3 (21.9)	10–35	0.0 (0.0)	0–0	100 (100)	$t_{(13)} = 24.0$, $P < 0.0001$
	23	29.4 (30.1)	22–41	0.0 (0.0)	0–0	100 (100)	$t_{(13)} = 42.5$, $P < 0.0001$
	30	21.9 (22.3)	15–28	0.1 (0.1)	0–1	99.6 (99.4)	$t_{(14)} = 27.6$, $P < 0.0001$
	37	29.6 (30.5)	17–40	0.0 (0.0)	0–0	100 (100)	$t_{(13)} = 39.5$, $P < 0.0001$
Estudio 3	2	16.6 (17.6)	11–36	0.0 (0.0)	0–0	100 (100)	$t_{(7)} = 23.6$, $P < 0.0001$
	9	14.2 (20.6)	0–41	0.0 (0.0)	0–0	100 (100)	$t_{(7)} = 6.5$, $P = 0.0003$
	16	21.3 (22.1)	13–33	0.0 (0.0)	0–0	100 (100)	$t_{(7)} = 30.8$, $P < 0.0001$
	23	26.8 (27.8)	18–41	0.0 (0.0)	0–0	100 (100)	$t_{(7)} = 34.5$, $P < 0.0001$
	30	31.8 (32.1)	25–39	0.0 (0.0)	0–0	100 (100)	$t_{(7)} = 70.1$, $P < 0.0001$
	37	19.2 (21.3)	9–36	0.0 (0.0)	0–0	100 (100)	$t_{(7)} = 17.7$, $P < 0.0001$

Abreviatura: na, no aplicable debido a infestaciones insuficientes en los perros control

Tabla 2 Media geométrica (aritmética) de conteos de garrapatas vivas *Rhipicephalus sanguineus*

	Día	Control no tratado		Lotilaner			Comparación
		**Media**	**Rango**	**Media**	**Rango**	**Media geométrica (aritmética) de eficacia (%)**	
Estudio 2	2	25.5 (27.6)	7–36	0.0 (0.0)	0–0	100 (100)	$t_{(13)} = 19.1$, $P < 0.0001$
	9	25.1 (26.0)	15–40	0.0 (0.0)	0–0	100 (100)	$t_{(14)} = 32.0$, $P < 0.0001$
	16	22.9 (24.5)	9–36	0.1 (0.3)	0–2	99.4 (99.0)	$t_{(14)} = 15.1$, $P < 0.0001$
	23	28.6 (29.9)	19–47	0.1 (0.1)	0–1	99.7 (99.6)	$t_{(14)} = 23.9$, $P < 0.0001$
	30	21.9 (23.4)	10–35	0.1 (0.1)	0–1	99.6 (99.5)	$t_{(14)} = 18.7$, $P < 0.0001$
	37	26.1 (28.1)	13–45	0.0 (0.0)	0–0	100 (100)	$t_{(13)} = 23.3$, $P < 0.0001$
Estudio 3	2	5.2 (6.5)	2–20	0.0 (0.0)	0–0	na	Na
	9	14.3 (16.0)	4–27	0.1 (0.1)	0–1	99.4 (99.2)	$t_{(7)} = 12.7$, $P < 0.0001$
	16	29.5 (31.0)	14–43	0.0 (0.0)	0–0	100 (100)	$t_{(7)} = 27.8$, $P < 0.0001$
	23	26.5 (27.8)	17–42	0.0 (0.0)	0–0	100 (100)	$t_{(7)} = 29.2$, $P < 0.0001$
	30	19.6 (20.4)	12–31	0.0 (0.0)	0–0	100 (100)	$t_{(7)} = 28.4$, $P < 0.0001$
	37	14.8 (15.3)	10–25	0.0 (0.0)	0–0	100 (100)	$t_{(7)} = 31.5$, $P < 0.0001$
Estudio 4	2	34.2 (35.4)	17–45	0.0 (0.0)	0–0	100 (100)	$t_{(4)} = 37$, $P < 0.0001$
	9	33.6 (34.4)	24–43	0.0 (0.0)	0–0	100 (100)	$t_{(4)} = 49.6$, $P < 0.0001$
	16	30.7 (31.4)	19–42	0.0 (0.0)	0–0	100 (100)	$t_{(4)} = 42.8$, $P < 0.0001$
	23	29.9 (30.5)	20–37	0.0 (0.0)	0–0	100 (100)	$t_{(4)} = 51.9$, $P < 0.0001$
	30	28.4 (29.5)	16–39	0.0 (0.0)	0–0	100 (100)	$t_{(4)} = 36.2$, $P < 0.0001$
	37	27.2 (28.3)	15–37	0.1 (0.1)	0–1	99.7 (99.6)	$t_{(4)} = 33.5$, $P < 0.0001$

Abreviatura: na, no aplicable debido a infestaciones insuficientes en los perros control

Tabla 3 Media geométrica (aritmética) de conteos de garrapatas vivas *Amblyomma americanum*

	Día	Control no tratado		Lotilaner			Comparación
		**Media**	**Rango**	**Media**	**Rango**	**Media geométrica (aritmética) de eficacia (%)**	
Estudio 5	2	18.1 (18.8)	12–28	0.0 (0.0)	0–0	100 (100)	$t_{(7)} = 29.3$, $P < 0.0001$
	9	16.5 (17.8)	9–29	0.1 (0.1)	0–2	99.1 (98.6)	$t_{(7)} = 18.4$, $P < 0.0001$
	16	18.0 (18.6)	12–24	0.0 (0.0)	0–0	100 (100)	$t_{(7)} = 29.1$, $P < 0.0001$
	23	16.0 (16.4)	12–23	0.0 (0.0)	0–0	100 (100)	$t_{(7)} = 35.6$, $P < 0.0001$
	30	16.3 (16.5)	12–20	0.0 (0.0)	0–0	100 (100)	$t_{(7)} = 53.3$, $P < 0.0001$
Estudio 6	2	19.4 (20.0)	14–34	0.0 (0.0)	0–0	100 (100)	$t_{(9)} = 38.8$, $P < 0.0001$
	9	16.2 (17.5)	5–27	0.1 (0.1)	0–1	99.0 (98.9)	$t_{(9)} = 18.3$, $P < 0.0001$
	16	16.9 (18.8)	5–36	0.0 (0.0)	0–0	100 (100)	$t_{(9)} = 18.0$, $P < 0.0001$
	23	18.1 (18.7)	10–24	0.0 (0.0)	0–0	100 (100)	$t_{(9)} = 35.0$, $P < 0.0001$
	30	18.3 (19.3)	8–27	0.0 (0.0)	0–0	100 (100)	$t_{(9)} = 26.6$, $P < 0.0001$

Tabla 4 Media geométrica (aritmética) de conteos de garrapatas vivas *Ixodes scapularis*

	Día	Control no tratado		Lotilaner			Comparación
		**Media**	**Rango**	**Media**	**Rango**	**Media geométrica (aritmética) de eficacia (%)**	
Estudio 7	2	26.3 (27.5)	20–44	0.0 (0.0)	0–0	100 (100)	$t_{(6)} = 24.7$, $P < 0.0001$
	9	13.8 (15.1)	4–23	0.1 (0.1)	0–1	99.3 (99.2)	$t_{(14)} = 13.4$, $P < 0.000$
	16	18.9 (19.6)	12–27	0.3 (0.5)	0–3	98.4 (97.5)	$t_{(6)} = 9.8$, $P < 0.0001$
	23	27.1 (27.8)	20–38	0.0 (0.0)	0–0	100 (100)	$t_{(6)} = 37.0$, $P < 0.0001$
	30	21.0 (21.3)	17–28	0.1 (0.1)	0–1	99.6 (99.4)	$t_{(14)} = 28.6$, $P < 0.0001$
	37	27.0 (28.0)	14–38	0.2 (0.3)	0–1	99.3 (99.1)	$t_{(6)} = 18.5$, $P < 0.0001$
Estudio 8	2	18.4 (18.6)	14–24	0.0 (0.0)	0–0	100 (100)	$t_{(7)} = 51.0$, $P < 0.0001$
	9	14.6 (15.0)	9–21	0.0 (0.0)	0–0	100 (100)	$t_{(7)} = 30.4$, $P < 0.0001$
	16	11.8 (12.8)	7–21	0.0 (0.0)	0–0	na	na
	23	13.6 (13.9)	10–19	0.0 (0.0)	0–0	100 (100)	$t_{(7)} = 37.9$, $P < 0.0001$
	30	10.6 (13.8)	0–20	0.0 (0.0)	0–0	100 (100)	$t_{(7)} = 6.9$, $P = 0.0002$
	37	14.1 (14.3)	11–17	0.0 (0.0)	0–0	100 (100)	$t_{(7)} = 55.1$, $P < 0.0001$

Abreviatura: na, no aplicable debido a infestaciones insuficientes en los perros control
